# Dataset from RNAseq analysis of differential gene expression in germinal vesicle and metaphase I stages of granulosa cells

**DOI:** 10.1016/j.dib.2025.111409

**Published:** 2025-02-18

**Authors:** Weijun Gong, Xiangqian Meng, Ping Lei, Penghao Li, Jing Li, Yan Zhu

**Affiliations:** a School of Mathematics Kunming University, Kunming University, Kunming 650214, China; b Jinxin Research Institute for Reproductive Medicine and Genetics, Sichuan Jinxin Xi'nan Women's and Children's Hospital, Chengdu 610000, China; c Yunnan Jinxin Jiuzhou Hospital, Kunming, 650233, Yunnan, China; d Engineering Research Center for Urban Modern Agriculture of Higher Education in Yunnan Province, School of Agriculture and Life Sciences, Kunming University, Kunming 650214, China

**Keywords:** Transcriptomics, Granulosa cells, Germinal vesicle, Metaphase I

## Abstract

We present transcriptomic data on human granulosa cells, which are essential parts of reproductive biology. These data consisted of single-cell transcriptomic data from 12 biological samples from two developmental stages (germinal vesicle and metaphase I). We evaluated these data by calculating gene expression profiles at different developmental stages and detected differences in gene expression in granulosa cells at the different developmental stages. We further evaluated the transcriptomic data via genomes from gene banks. Thus, these new data demonstrate the feasibility of obtaining reliable transcriptomic data from individual samples at different developmental stages, and provide a framework for systematic transcriptomic studies of granulosa cell development.

Specifications Table

**Table:** 

Subject	Biological sciences/Omics: Transcriptomics
Specific subject area	Descriptive work on the transcriptome of granulosa cells in two stages and their associated gene expression differences.
Data format	Clean and analysed
Type of data	Tables
Data collection	These data were sequenced from granulosa cells (GCs) collected from two patients. RNA extraction followed a modified Smart-Seq2 protocol, and sequencing was performed on the Illumina NovaSeq 6000, generating 150-bp paired-end reads. Transcriptome processing involved removing low-quality reads with FastQC v0.12.1, and aligning high-quality reads to the GRCh38 reference genome using Tophat2 v2.1.0. Gene expression levels were quantified with featureCounts v2.0.1, and differential expression analysis was conducted using DESeq2 in R v4.2.0. GO and KEGG pathway enrichment analyses were performed with clusterProfiler and KEGG.db. Visualization of clustering and gene relationships used t-SNE (Rtsne) and heatmap generation with pheatmap.
Data source location	Granulosa cell specimens were collected in November 2023 from two 28-year-old female patients at Sichuan Jinxin Xinan Women and Children's Hospital (Bisheng Hospital, geographical coordinates 104.09, 30.59) in Sihuan, China. Single-cell cDNA libraries were prepared from the ovarian samples to study gene expression in human granulosa cells.
Data accessibility	Repository name: NCBI Sequence Read Archive (SRA) Data identification number: BioProject ID PRJNA1191901 Reference transcriptome for the target human: GRCh38.p14 (GCA_000001405.29) BioSamples: SAMN45096099–SAMN45096110 (Smart-seq2 data) Direct URL to data: https://www.ncbi.nlm.nih.gov/bioproject/?term=PRJNA1191901

## 1 Value of the Data

• These data provide valuable genomic resources for human granulosa cells, which are critical for human reproduction and have been subject to limited sequencing efforts regarding their genomes and transcriptomes.
• These data will be of particular interest to researchers focused on transcriptional regulation, cell‒cell interactions, and developmental biology, especially those investigating the distinct transcriptional processes and transcription factor networks of human granulosa cells during key developmental stages (germinal vesicle and metaphase I stages).
• These data can be used as a foundation for future systematic transcriptomic analyses of human granulosa cells at these critical developmental phases, facilitating the characterization of developmental markers and enhancing our understanding of ovarian biology.

## 2 Background

Human folliculogenesis poses significant challenges in reproductive biology, requiring intricate coordination between oocyte development and the growth of surrounding granulosa cells (GCs) [1,2]. This situation emphasizes the pressing need for advanced methodologies to study these complex biological processes. Recent advancements in transcriptomic technologies, particularly next-generation sequencing, have initiated a transformative shift in understanding the molecular mechanisms governing follicular development, providing promising avenues for in-depth analysis of gene expression dynamics. Prior studies have highlighted the importance of GCs in human fertility, revealing their critical roles in hormone synthesis and oocyte maturation, thereby highlighting the necessity of detailed investigations into these cells [[3], [4], [5]]. Furthermore, datasets from previous studies, that encompassed various developmental stages of GCs, have demonstrated the effectiveness of transcriptomic analyses in elucidating the regulatory networks involved in folliculogenesis [6,7]. Building on these foundations, our study focuses specifically on the transcriptomic landscape of granulosa cells at the germinal vesicle (GV) and metaphase I (MI) stages, aiming to provide a comprehensive reference for understanding transcriptional regulation during these critical periods. By utilizing real-world data rather than synthetic datasets, our research aims to increase the accuracy and reliability of findings, thereby contributing to advancements in reproductive health and fertility treatments.

## 3 Data Description

The dataset is available in the NCBI Sequence Read Archive (SRA) under BioProject IDPRJNA1191901, which includes all clean sequence data (https://www.ncbi.nlm.nih.gov/bioproject/?term=PRJNA1191901).

(1) Reference transcriptomes for the target species: GRCh38.p14 (GCA_000001405.29).
(2) Smart-seq2 data for the 12 samples from the germinal vesicle (GV) and metaphase I (MI) stages: BioSamplesSAMN45096099_SAMN45096110.

**Fig. 1:**
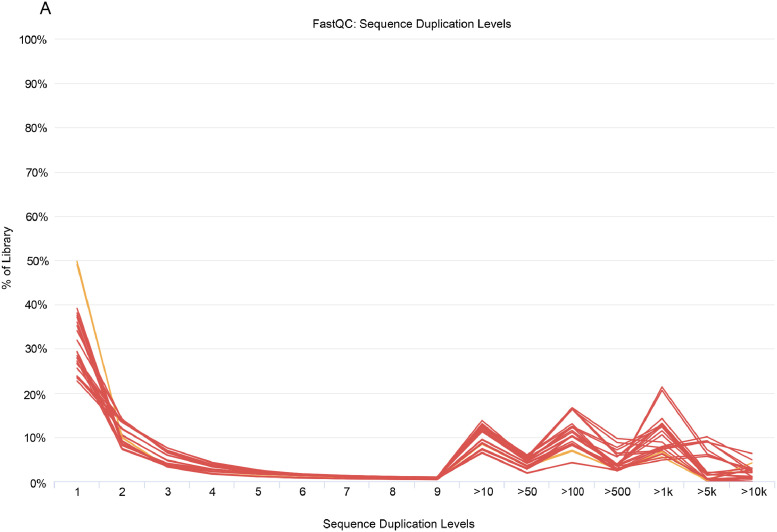
Distribution of sequence duplication levels.

**Fig. 2:**
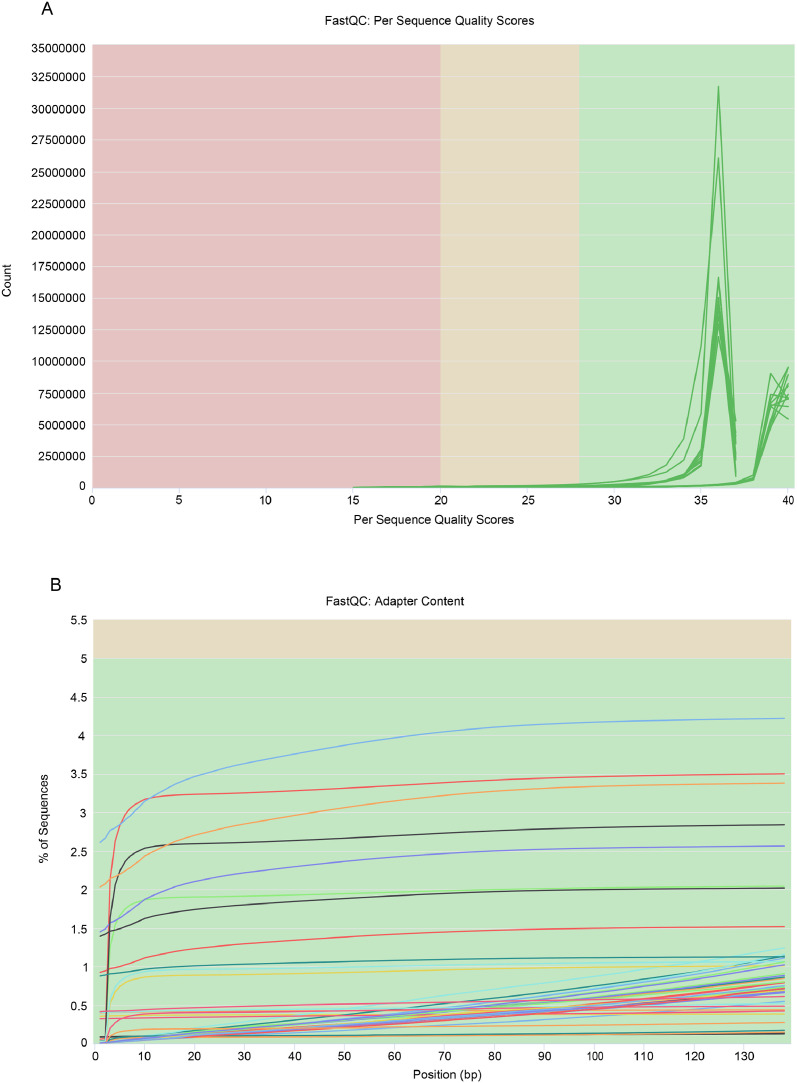
(A) Per sequence quality scores and (B) adapter content detection.

The dataset archived in the NCBI SRA includes sequencing data for human granulosa cells, detailed across multiple samples. The sequencing statistics, summarized in Table 1, indicate an average of 21,442,076 sequences per biosample, with total bases averaging 3.16 billion. The average GC content was 44 %, and high Q20 and Q30 scores underscore the sequencing quality and data reliability. This dataset provides essential insights into the molecular profile of human granulosa cells. The consistency in the GC content across samples supports robust downstream analyses, whereas the overall sequencing quality highlights the data's suitability for detailed gene expression studies. These metrics underscore the dataset's utility in understanding gene expression patterns involved in granulosa cell development. For further details on sequencing quality and sample-specific metrics, refer to Table 1. Table 2 summarizes the number of transcripts detected and the mapped read percentage for each sample. Sequencing data showed a mapped read percentage ranging from 77 % to 94 %, highlighting high alignment quality. As shown in Fig. 1, the sequence duplication levels are primarily concentrated at low levels (1–2 duplicates), indicating excellent library complexity and uniformity, meeting the standards for high-quality transcriptome sequencing. FastQC analysis further confirmed the high sequence quality, with median Phred scores above 30 across all samples and minimal adapter contamination (< 5 %), as shown in Fig. 2.

**Table 1:** Sequencing quality metrics for human granulosa cells at the GV and MI stages.

Biosample	Total Sequences	Total Bases (Gbp)	GC content	Q30
SAMN45096099	23,729,968	3.50	0.43	0.96
SAMN45096106	21,512,573	3.20	0.46	0.97
SAMN45096100	23,945,755	3.50	0.49	0.97
SAMN45096101	21,199,690	3.10	0.45	0.96
SAMN45096102	14,338,339	2.10	0.45	0.96
SAMN45096103	16,972,728	2.50	0.46	0.97
SAMN45096104	15,529,784	2.20	0.41	0.97
SAMN45096105	47,619,431	7.10	0.42	0.96
SAMN45096110	20,985,715	3.10	0.42	0.96
SAMN45096107	19,018,793	2.80	0.43	0.97
SAMN45096108	16,886,200	2.50	0.40	0.97
SAMN45096109	15,565,930	2.30	0.50	0.97

**Table 2:** Summary of Detected Transcripts and Mapped Read Percentage per Sample.

Biosample	Sample	transcripts	mapped read percentage
SAMN45096099	BS-2–0107	29,741	90.06 %
SAMN45096106	BS-2–1128	26,655	89.50 %
SAMN45096100	BS-2–0208	21,434	88.40 %
SAMN45096101	BS-2–0218	26,109	90.19 %
SAMN45096102	BS-2–0220	45,244	77.16 %
SAMN45096103	BS-2–0303B	37,313	80.66 %
SAMN45096104	BS-2–0308A	33,341	88.56 %
SAMN45096105	BS-2–0403	39,670	81.43 %
SAMN45096110	DS-2–1205	29,481	83.62 %
SAMN45096107	DS-2–0215	28,791	82.11 %
SAMN45096108	DS-2–0310	22,098	93.39 %
SAMN45096109	DS-2–0418	25,650	88.20 %

## 4 Experimental Design, Materials and Methods

### 4.1 Isolation of human granulosa cells (GCs)

Granulosa cells were obtained from two patients for single-cell cDNA library preparations aimed at studying gene expression in human granulosa cells. The specimens were collected in November 2023 from two 28-year-old female patients at Sichuan Jinxin Xinan Women and Children's Hospital (Bisheng Hospital, geographical coordinates 104.09, 30.59) in Sichuan, China. After oocyte retrieval under B-ultrasound guidance, the cumulus‒oocyte complex (COC) was transferred via a pipette into well 1 of a four-well plate, which contained 300 µL of 80 U/mL hyaluronidase (ART-4007-A, SAGE, USA), 300 µL of G-MOPSTM PLUS (10,130, Vitrolife, Sweden), and 500 µL of mineral oil (ART-4008–5P, SAGE, USA). The COC was aspirated repeatedly until the granulosa cells detached from the oocyte, ensuring that the aspiration time did not exceed 30 s. The oocytes were then moved to well 2, which contained 500 µL of G-MOPSTM PLUS and 500 µL of mineral oil, and rinsed 3–4 times before being transferred to well 3. Well 3 also contained 500 µL of G-MOPSTM PLUS and 500 µL of mineral oil. Once the granulosa cells were separated, the oocytes were removed, and all the granulosa cells were collected in a 200 µL tube.

### 4.2 Construction and analysis of a single-cell cDNA library from granulosa cells

Eight randomly selected granulosa cells from the germinal vesicle stage and four randomly selected granulosa cells from the metaphase I stage were quickly transferred into lysis buffer via a mouth pipette. We then performed single-cell transcriptome amplification with a modified Smart-Seq2 protocol. Each cell was briefly placed in cell lysis buffer that contained 0.1 µL of RNase inhibitor (Clontech), 1.9 µL of 1 % Triton X-100 solution, 1 µL of a 10 mM dNTP mixture, and 1 µL of oligo-dT primer at 5 µM. Reverse transcription was initiated with 0.5 µL of SuperScript II reverse transcriptase (200 U/µL, Invitrogen), 0.25 µL of RNase inhibitor (40 U/µL, Clontech), 2 µL of Superscript II first-strand buffer (5 ×, Invitrogen), 0.5 µL of DTT (0.1 M, Invitrogen), 2 µL of Betain (5 M, Sigma), 0.06 µL of MgCl2 (1 M, Sigma), and 0.1 µL of TSO (100 µM). The mixture was incubated at 25 °C for 5 min, 42 °C for 60 min, 50 °C for 30 min, and finally, 72 °C for 10 min. For PCR preamplification, 22 cycles of KAPA HiFi HotStart Ready MIX (KAPA Biosystems) were used; the IS PCR primer concentration was adjusted to 50 nM (first, 4 cycles at 98 °C for 20 s, 65 °C for 30 s, and 72 °C for 5 min; then 18 cycles at 98 °C for 20 s, 67 °C for 15 s, and 72 °C for 5 min, ending at 72 °C for 5 min). The amplified samples were purified twice via 0.8X AMPure XP beads (Beckman, A63882). A library was created from the enriched cDNA fragments by attaching them to C1 beads, via KAPA Hyper Prep Kits (KK8504), with ligation via an NEB U-shaped adaptor. Sequencing was performed on the Illumina NovaSeq 6000 platform to generate 150-bp paired-end reads.

Raw RNA-Seq reads containing 10 % or more low-quality bases (https://github.com/1787308709/RNAseq-Cleaning-and-DDS-Processing), as well as adapters and experimental artefacts (including UP1, UP2, and polyA sequences), were removed via custom scripts. The resulting high-quality reads were then aligned to the GRCh38 reference genome with TopHat2 (v2.1.0 [8]) via default parameters. To quantify the transcription levels of the annotated genes, Cufflinks (v2.2.1 [9]) was subsequently employed.

### 4.3 Gene expression analysis

Transcript abundance differences were identified via FastQC (v0.12.1 [10]) for quality control, where low-quality reads and contaminants were removed to obtain clean reads. Sequence metrics, including length, base composition, base quality, and GC content, were evaluated for both raw and cleaned data. Gene expression levels from aligned BAM files were quantified via featureCounts (v2.0.1 [11]), yielding gene counts that were normalized by TPM. Differential expression analysis was performed via DESeq2 [12] in R (v4.2.0 [13]), a DDS matrix was constructed (https://github.com/1787308709/RNAseq-Cleaning-and-DDS-Processing), and the data were normalized to identify genes with significant expression changes. For sample clustering, t-SNE plots were generated with the R package Rtsne, via differential gene analysis. The relationships among differential genes involved were visualized via box, violin, and volcano plots generated via gridExtra and ggplot2 in R. Gene Ontology (GO) enrichment analysis was conducted with clusterProfiler, revealing insights into molecular functions, cellular components, and biological processes. KEGG pathway enrichment analysis was performed with KEGG.db, which lists pathways enriched in differential genes. Additionally, pheatmap was used to create heatmaps via Pearson correlation to better visualize sample clustering (Figure S1).

## Limitations

Not applicable.

## Ethics Statement

This study adhered to the principles outlined in the Declaration of Helsinki and received approval from the Ethics Committee for Reproductive Medicine at Sichuan Jinxin Xinan Women's and Children's Hospital on December 19, 2023 (protocol reference: 2023 Medical Ethics Review No. 035).

## CRediT Author Statement

**Weijun Gong:** Investigation, Data collection, Methodology, Super Vision, Visualization, Writing – original draft, Writing – review & editing; **Xiangqian Meng:** Conceptualization, Methodology, Supervision, Project administration, Validation, Writing – original draft, Writing – review & editing; **Ping Lei:** Investigation, Data collection, Methodology, Writing – original draft, Writing – review & editing; **Jing Li:** Investigation, Data collection, Writing – original draft, Writing – review & editing; **Penghao Li**: Conceptualization, Methodology, Supervision, Visualization, Project administration, Validation; **Yan Zhu:** Conceptualization, Methodology, Supervision, Project administration, Validation.

## Data Availability

National Center for Biotechnology InformationSingle-cell cDNA library data for granulosa cells (Original data).

## Appendix Supplementary materials

Image, application 1
