# Supplementary figures and images for: Dataset from RNAseq analysis of differential gene expression in germinal vesicle and metaphase I stages of granulosa cells
Source: Data Brief. 2025 Feb 18;59:111409. doi: 10.1016/j.dib.2025.111409 (PMC11919372; doi:10.1016/j.dib.2025.111409)

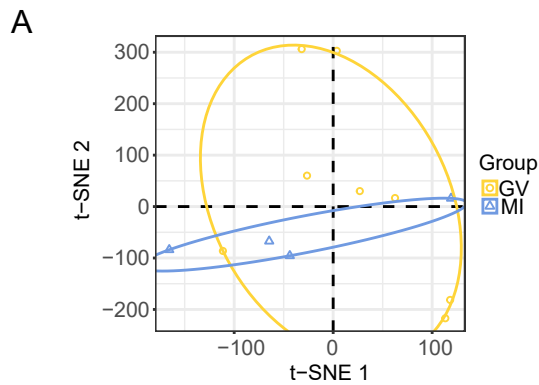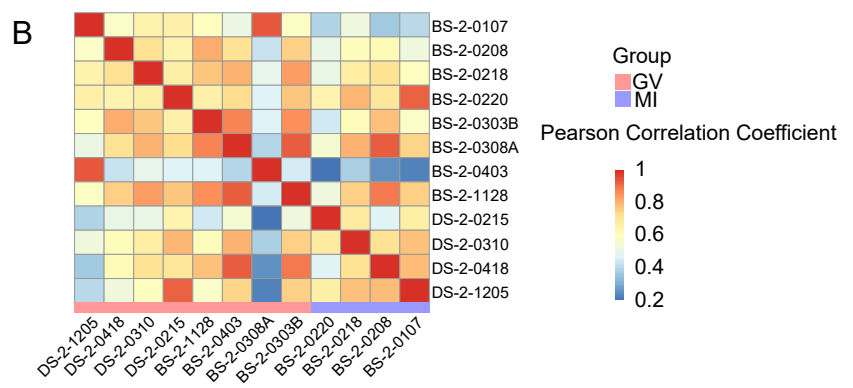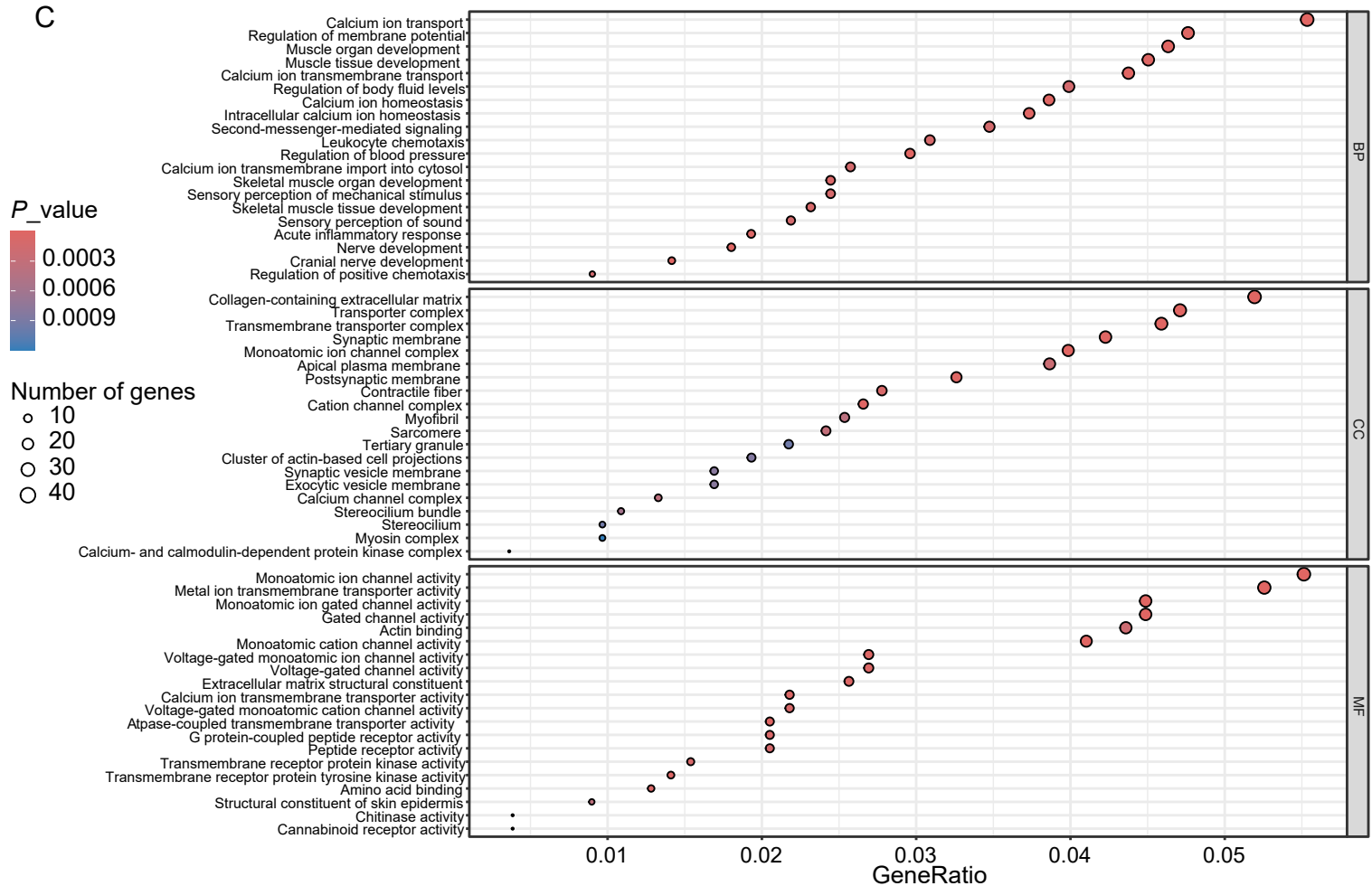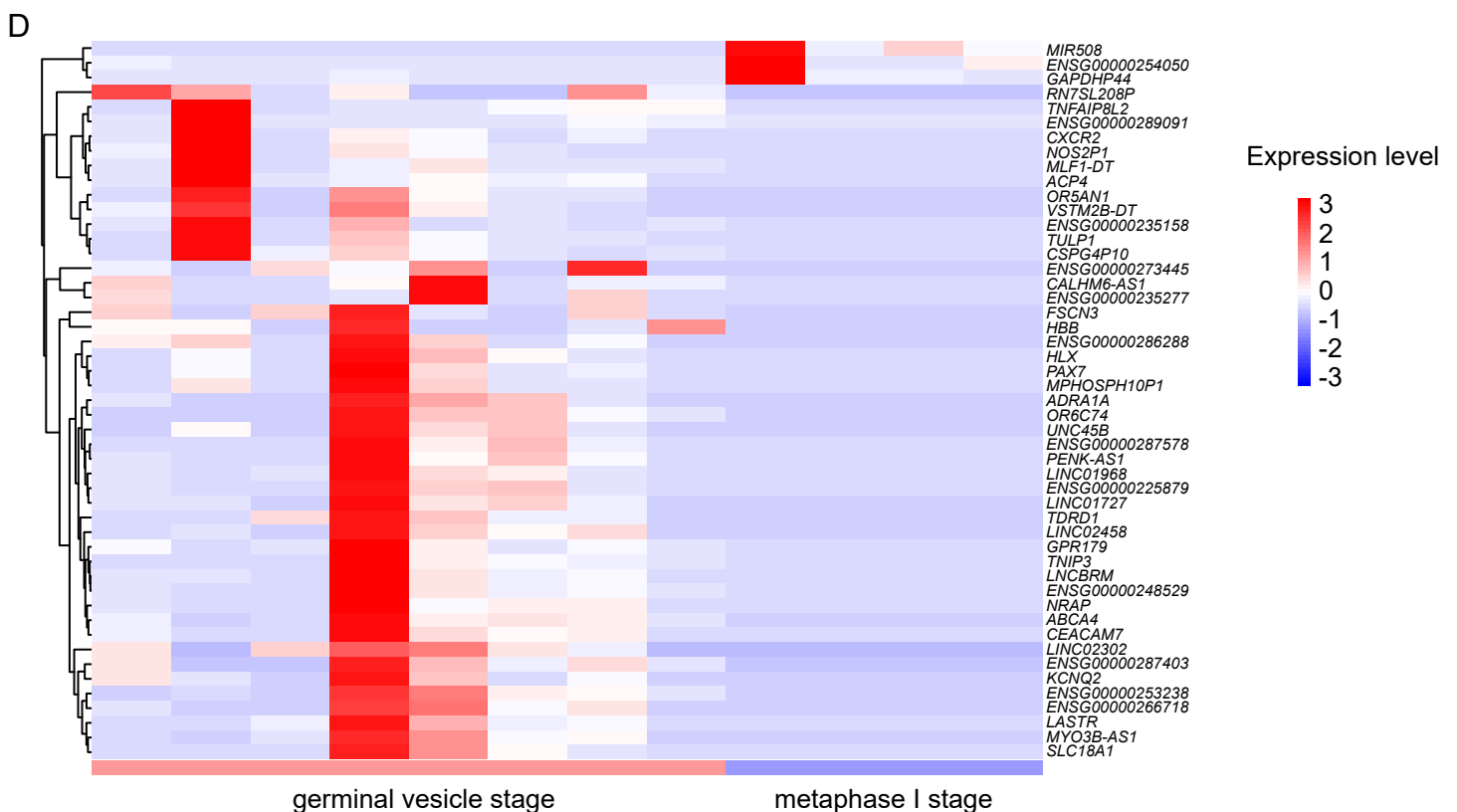

Supplement: Supplementary file 1 [file mmc1.pdf]
